# Hydrodynamic stress stimulates growth of cell clusters via the ANXA1/PI3K/AKT axis in colorectal cancer

**DOI:** 10.1038/s41598-019-56739-7

**Published:** 2019-12-27

**Authors:** Takeshi Hagihara, Jumpei Kondo, Hiroko Endo, Masayuki Ohue, Yoshiharu Sakai, Masahiro Inoue

**Affiliations:** 1 Department of Clinical Bio-resource Research and Development, Graduate School of Medicine, Kyoto University, Yoshida-honmachi, Sakyo-ku, Kyoto 606-8501 Japan; 2 Division of Gastrointestinal Surgery, Department of Surgery, Graduate School of Medicine, Kyoto University, Yoshida-honmachi, Sakyo-ku, Kyoto 606-8501 Japan; 3 Department of Biochemistry, Osaka International Cancer Institute, 3-1-69, Otemae, Chuo-ku, Osaka 541-8567 Japan

**Keywords:** Biophysics, Colorectal cancer

## Abstract

Cancer cells are exposed to various stresses *in vivo*, including hydrodynamic stress (HDS). HDS on cancer cells in the blood stream can influence the metastatic potential. Recent studies revealed that circulating tumor cell clusters are more responsible for metastasis than circulating single cells. Nevertheless, most studies on HDS are based on single cells prepared from established cancer cell lines. Here, we used cancer tissue-originated spheroids (CTOS) as a patient-derived, 3D organoid model to investigate the effect of HDS on cancer cell clusters. We found that HDS induced the growth of cancer cell clusters in a population of colorectal CTOSs. Microarray analyses revealed that the multifunctional protein, Annexin 1 (ANXA1), was upregulated upon HDS exposure. Chemically-induced membrane damage also triggered the expression of ANXA1. A knockdown of *ANXA1* revealed that ANXA1 regulated HDS-stimulated growth in colorectal CTOSs. Mechanistically, activating the PI3K/AKT pathway downstream of ANXA1 contributed to the phenotype. These findings demonstrate that HDS induces the growth of cancer cell clusters via ANXA1/PI3K/AKT axis, which helps to elucidate the pro-metastatic feature of circulating cancer cell clusters.

## Introduction

In the blood stream, cancer cells endure various stresses. In addition to the loss of the cell-matrix interaction, which assures anchorage-dependent survival^1^, blood flow incurs various levels of hydrodynamic stress (HDS) on cells. HDS, including fluid shear stress, affects cell survival^2,3^, proliferation^4,5^, stemness^6^, chromosomal instability^7^, and metastasis potential^8^, either positively or negatively, possibly depending on the context. Fluid shear stress is the most well-defined, well-studied type of HDS. Fluid shear force acts on an object in a system where the fluid is moving across a solid surface. Reportedly, fluid shear stress suppressed the growth of a colorectal cancer (CRC) cell line^4^ and induced apoptosis in various cell lines^3^. Another report suggested that fluid shear stress promoted the invasion of a prostate cancer cell line, via the activation of Yes-Associated Protein (YAP)^9^. Those studies are examples of how the biology of cancer cell responses to HDS is increasingly attracting attention. Additional advances that have contributed to this field included the development of microfluidic devices^2,6,10^. In contrast, the cellular model used for investigating cancer responses to HDS is yet to be improved. Most HDS studies have been performed with either 2D adherent cells or single cells of established cell lines in suspension. However, neither the 2D cell sheet nor the single cell suspension has recapitulated the way that viable cancer cells exist in a physiological environment. Additionally, the use of established cell lines has limitations, due to the altered nature of cells, compared to cells in the original tumor^11^. On the other hand, recently, 3D organoid cultures were introduced with cancer cells derived from patient samples. This approach provided a more physiological cellular model that could better reflect the characteristics of original tumors *in vivo*^11^. We have developed a primary culture method for preparing cancer cell organoids from tumor tissue, called cancer tissue-originated spheroids (CTOSs); in this setting, cell-cell contact was maintained throughout the preparation and culture processes. We previously reported that CTOSs were stable in suspension^12,13^. On the other hand, dispersed single primary cancer cells were prone to anoikis, a type of apoptosis induced by the loss of cell-matrix or cell-cell interactions^1,12^; in contrast, established cell lines are relatively resistant to anoikis.

Recent advances in the field of circulating tumor cells (CTCs) suggested that cancer cells are highly viable in the blood stream, when they form cell clusters, rather than single cells^14^. Thus, cancer cell clusters are more likely the origin of metastasis^15^. Despite these advances in cellular models and the understanding of the importance of cell clusters for cancer cell viability in the blood stream, the effect of HDS on cancer cell clusters remains to be fully investigated.

Recently, we reported that disrupting the architecture of cell clusters with strong HDS treatment activated both stemness and growth in CRC CTOSs, via the activation of the WNT pathway^13^. However, in that study, the HDS introduced to the cancer cell clusters was above physiological levels. Additionally, it remains to be elucidated whether the disruption of the cell cluster architecture is important, or whether HDS alone (without disrupting the cell cluster) caused the positive growth effects on cancer cell clusters. Therefore, in the present study, we investigated how ‘mild’ HDS, which did not disrupt the 3D architecture of cancer cell clusters, affected cancer cell cluster growth. We found that mild HDS was sufficient to stimulate growth in CRC cell clusters, via Annexin 1 (ANXA1) expression, induced by plasma membrane damage. The mechanism involved HDS induction of ANXA1, which activated the PI3K/AKT pathway. These findings will help to elucidate the pro-metastatic feature of circulating cancer cell clusters.

## Results

### Hydrodynamic stress stimulates growth of cancer cell clusters in some colorectal cancer CTOS lines

**Figure 1:**
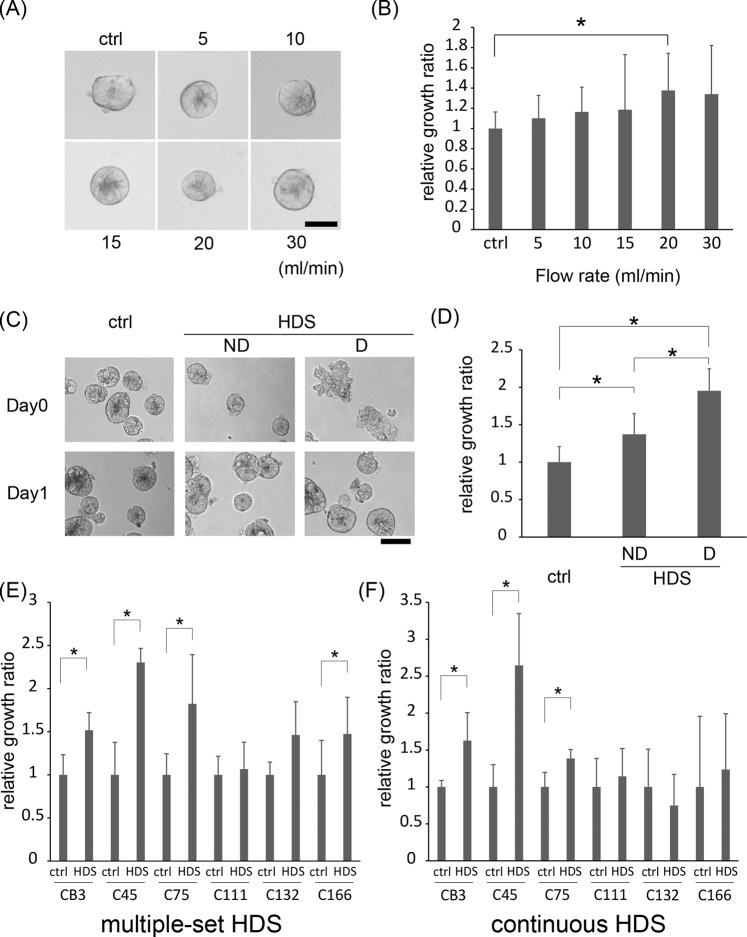
Hydrodynamic stress (HDS) stimulates growth of some colorectal cancer tissue-originated spheroids (CTOSs). **(A**) Phase-contrast images of C45 CTOSs immediately after exposure to a single-set pulse HDS (4 syringe-passings). Images are representative of each flow rate, as indicated. Scale bar: 50 μm **(B)**. Relative growth of C45 CTOS evaluated by ATP assay, 7 days after single-set pulse HDS (4 syringe-passings) delivered at different flow rates. The values are the average ± SD, normalized to the non-pulsed control (ctrl). N = 10 for each condition; *P < 0.05. (**C**) Phase-contrast images of C45 CTOSs immediately after exposure (day 0) to HDS (30 ml/min, 6 syringe-passings) and 1 day later (day 1). Representative images show CTOSs with non-disrupted (ND) and disrupted (D) architectures. Scale bar: 100 μm. **(D**) Relative growth of C45 CTOS evaluated by ATP assay, with or without disruption morphology, 7 days after a single-set pulse HDS. The values are the average ± SD, normalized to ctrl. N = 20 for each condition. *P < 0.01. **(E**) Relative growth of various CTOS lines evaluated by ATP assay, 14 days after multiple-set pulse HDS. The values are the average ± SD, normalized to ctrl. N = 12 for each condition. *P < 0.05. **(F**) Relative growth of various CTOS lines evaluated by ATP assay, after 7 days of culture with continuous HDS. The values are the average ± SD, normalized to the static condition (ctrl). N = 6 for each condition. *P < 0.05.

Previously, we reported that CRC cell clusters exhibited enhanced growth and stemness restoration in response to a disruption of their architecture. In that study, we used CTOSs as the model for cell clusters^13^. In physiological conditions, the architecture of cell clusters, supported by cell-cell interactions, could be disturbed by different causes, such as tissue inflammation and physical forces, like hydrodynamic stress (HDS). In the previous study, we applied strong HDS to disrupt the cell cluster architecture of CTOSs. In the present study, to gain a better understanding of how HDS affected cell clusters, we first evaluated whether HDS alone, without an architectural disruption, might be sufficient to induce growth in CRC CTOSs. After single-set pulse HDS was applied at mild levels with the syringe loading method (Materials and Methods and Supplementary Fig. S1A), the morphology of most C45 CTOSs remained intact, with no architectural disruption (Figs. 1A and S2); nevertheless, the growth of C45 CTOS was stimulated by mild HDS (Figs. 1B and S3A). To confirm the effect of HDS on growth stimulation without disrupting CTOSs, we exposed C45 CTOSs to single-set pulse HDS (Supplementary Fig. S1A), and morphologically distinct two groups of CTOSs were transferred to 96-well plates, at 10 CTOSs per well; one group with apparent CTOS architecture disruptions and another without disruptions (Fig. 1C). After one week of culture, significant growth stimulation was observed in non-disrupted CTOSs after single-set pulse HDS, although less than disrupted CTOSs (Figs. 1D and S3B). Thus, we confirmed that HDS stimulated the growth of cell clusters, even without disrupting their architecture. Importantly, although the effect of a single-set pulse HDS was moderate, both multiple-set pulse HDS (Figs. 1E and S1B) and continuous HDS (Figs. 1F, S1C and S4) robustly stimulated the growth of C45 CTOSs. This phenomenon was observed in 4 out of 6 CRC CTOS lines (CB3, C45, C75, C111, C132, and C166) (Fig. 1E,F and Supplementary Table S2), which indicated that it was not a phenotype specific to an atypical CTOS line. These results support that HDS had a positive effect on the growth of cancer cell clusters. Of note, for dispersed single cells prepared from CTOSs, HDS did not either stimulate or suppress growth (Supplementary Fig. S5), which indicated that this phenomenon was only apparent with cancer cells in cell clusters.

### HDS-induced membrane damage triggers growth stimulation

**Figure 2:**
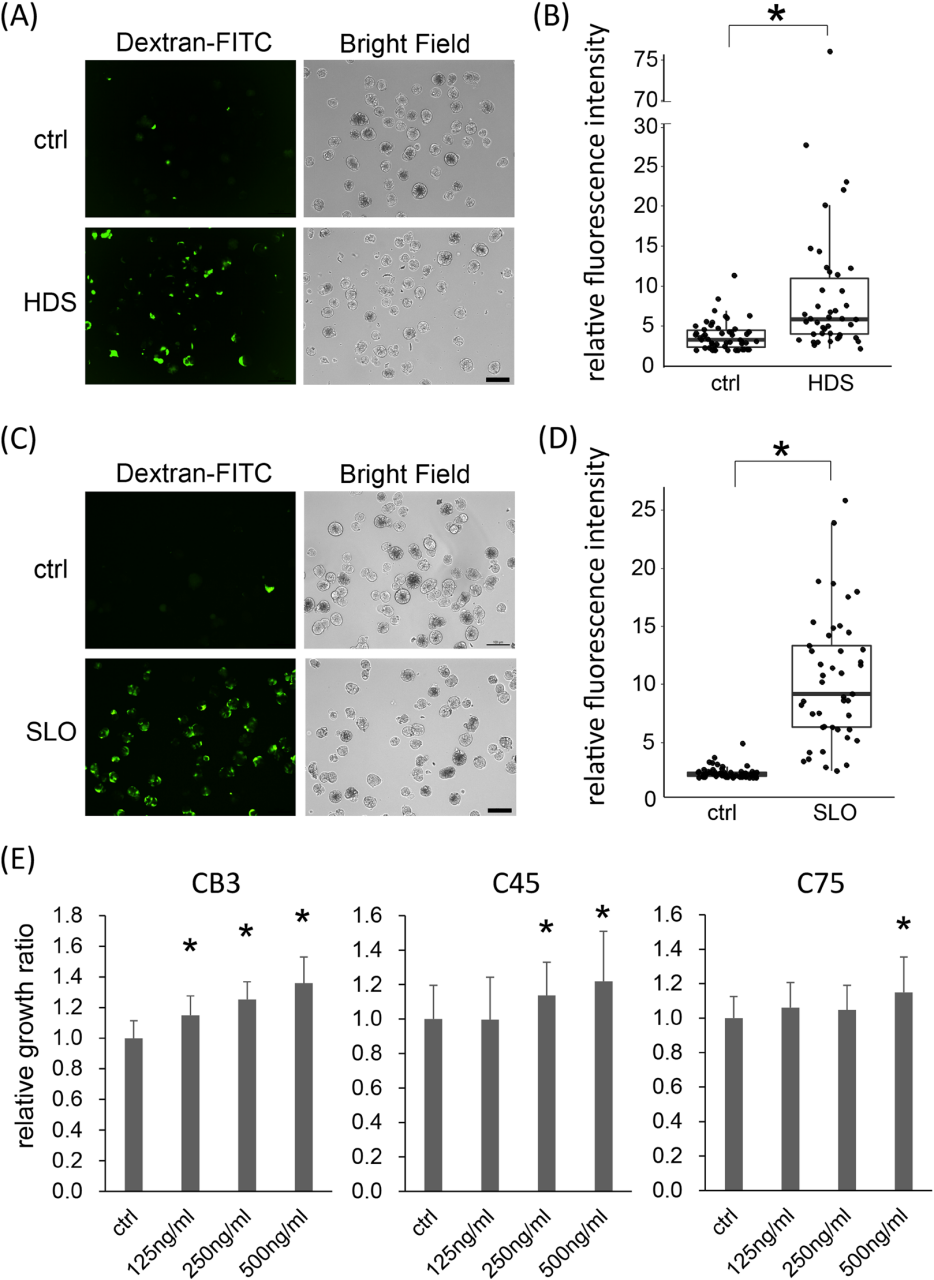
Hydrodynamic stress (HDS) induces membrane damage, which triggers growth stimulation in colorectal cancer tissue-originated spheroids (CTOSs). **(A**) Fluorescence and phase-contrast images of C45 CTOSs exposed to single-set pulse HDS, in the presence of FITC-conjugated dextran. Scale bar: 100 μm. **(B)** Quantified fluorescence intensity of CTOSs shown in panel (**A**). Data are the box-and-whisker dot plots; the boxes indicate inter-quartile range and the horizontal lines depict median, and the vertical lines show 1.5 times the inter-quartile range. Each dot is the intensity value of individual single CTOS, N = 51 and N = 42 for the control (ctrl) and HDS groups, respectively. Wilcoxon rank sum test was performed for the statistical analysis. *P < 0.05. (**C**) Fluorescence and phase-contrast images of C45 CTOSs treated with 125 ng/ml streptlysin-O (SLO) for 10 min in the presence of FITC-conjugated dextran. Scale bar: 100 μm. (**D**) Quantified fluorescence intensity of CTOSs shown in panel (**C**). Data are the box-and-whisker dot plots presented in the same fashion as (**B**). Each dot is the intensity value of individual single CTOS, N = 56 and N = 45 for control and SLO-treated groups, respectively. Wilcoxon rank sum test was performed for the statistical analysis. *P < 0.05. (**E**) Relative growth of CB3, C45, and C75 CTOSs evaluated by ATP assay, 7 days after treatment with different SLO concentrations. Values are the average ± SD, normalized to ctrl. N = 20 for each condition. *P < 0.05.

One of the robust effects of HDS on cells is the damage to the plasma membrane^16^. Small pores in the plasma membrane are reportedly generated by exposure to fluid shear stress, but these pores are rapidly repaired (within 5 s) by Ca^2+^-dependent vesicle-vesicle fusion events^17,18^. Therefore, next, we investigated whether the membrane damage induced by HDS in cell clusters might trigger growth stimulation. Cells with membrane damage were identified by FITC-conjugated dextran uptake, because FITC-dextran is only incorporated into the cytoplasm after the plasma membrane is damaged^19^. Indeed, we found that HDS induced FITC-dextran incorporation into CTOSs (Figs. 2A,B and S6). Next, we examined whether membrane damage alone might stimulate growth. CTOSs were chemically treated with streptlysin-O (SLO), a cholesterol-dependent pore-forming toxin, for 10 min to introduce membrane damage^19^ without applying HDS. SLO successfully induced membrane damage, evident by FITC-dextran incorporation (Figs. 2C,D and S6). After 7 days in culture, SLO treatment stimulated growth in 5 out of the 6 CTOS lines (Figs. 2E and S7). SLO did not stimulate the growth of the C132 CTOS line; however, that line did not exhibit stimulated growth in response to either multiple-set pulse HDS or continuous HDS (Fig. 1E,F). Taken together, these results indicated that HDS-induced membrane damage might trigger CTOS growth.

### HDS induces a distinct set of genes, including *ANXA1*, in cancer cell clusters

**Figure 3:**
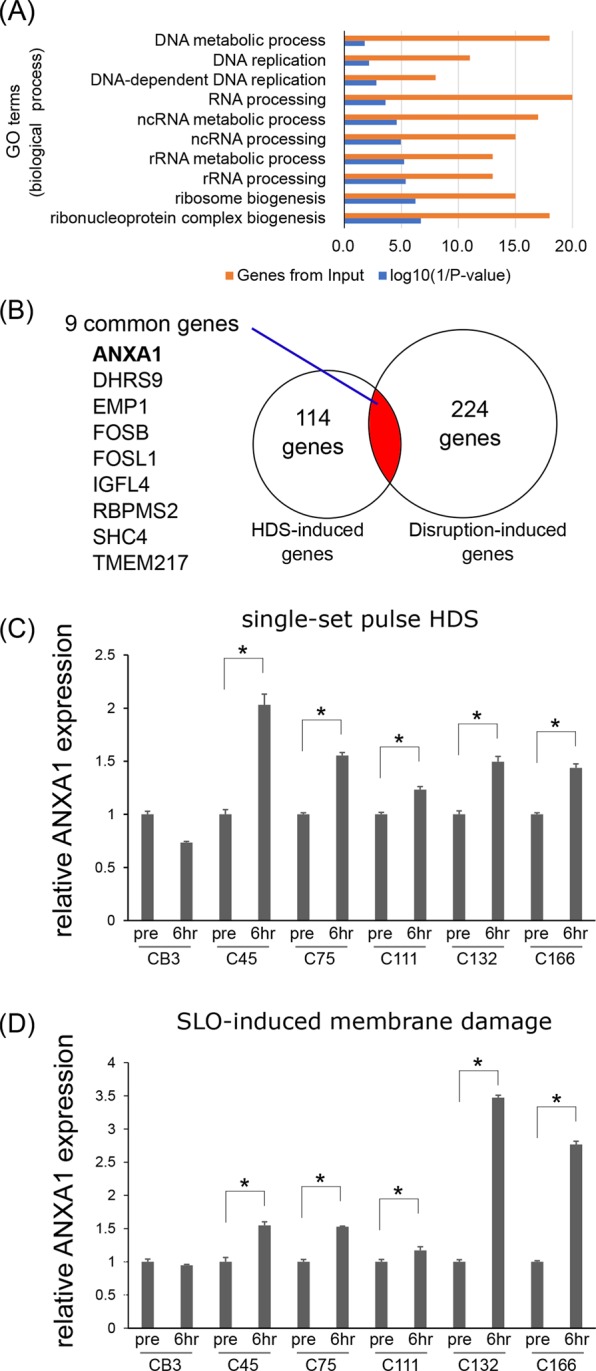
Hydrodynamic stress (HDS) induces upregulation of *Annexin 1* (*ANXA1*) in cancer cell clusters. (**A**) Gene ontology (GO) analysis of gene-expression microarray data for C45 CTOS treated with single-set pulse HDS. At 6 h after HDS, 123 genes were upregulated by more than 1.5-fold; these genes were analyzed for GO enrichment. Orange bars: the number of genes included in each GO term. Blue bars: the log10(1/P-value) for each GO term. **(B**) Venn diagram shows overlap of 9 genes that were upregulated, both by mild HDS (single-set pulse HDS), which did not disrupt cell membranes, and by strong HDS, which caused architectural disruptions^13^. **(C**) Relative expression of *Annexin 1* (*ANXA1*) mRNA before (pre) and 6 h after single-set pulse HDS, in multiple CTOS lines. Data are the average ± SD; N = 3 for each run. *P < 0.05. (**D**) Relative expression of ANXA1 mRNA, before and 6 h after SLO treatment in multiple CTOS lines. Data are the average ± SD; N = 3 for each run. *P < 0.05.

To investigate further the mechanism underlying HDS-stimulated growth, we performed a comprehensive gene expression analysis. After 6 h of HDS, we found that 123 genes were upregulated. A gene ontology (GO) analysis showed that 10 GO terms related to biological processes were enriched (Fig. 3A and Supplementary Table S3). Most of the enriched GO terms were related to DNA replication and RNA processing, which are involved in cell cycle progression. This result supported the finding that HDS stimulated the proliferation of cancer cells in cell clusters.

Next, we hypothesized that the genes responsible for this phenotype should be included in the gene sets that showed altered expression after strong HDS and mechanical disruption of the CTOS architecture^13^. Among the genes upregulated by mild HDS, 9 genes were also highly induced by strong HDS (Fig. 3B, Supplementary Table S3). One of these 9 genes, *ANXA1*, encoded a multifunction protein that contributes to membrane repair upon damage^20,21^. As shown above, membrane damage was involved in the growth induced by HDS. Thus, we focused on the role of ANXA1 in HDS-induced growth stimulation.

We evaluated the levels of *ANXA1* expression in multiple CTOS lines. In all CTOS lines, except CB3, *ANXA1* was upregulated within 6 h of the single-set pulse HDS (Fig. 3C) and the continuous HDS (Supplementary Fig. S8). This indicated that HDS induction of ANXA1 was a common phenomenon in CRC cell clusters. Importantly, membrane damage by SLO, in the absence of HDS, also induced ANXA1 expression in these 5 CTOS lines, but not in the CB3 line, as observed with HDS (Fig. 3D).

### ANXA1 regulates HDS-induced growth stimulation in a CRC cell population

**Figure 4:**
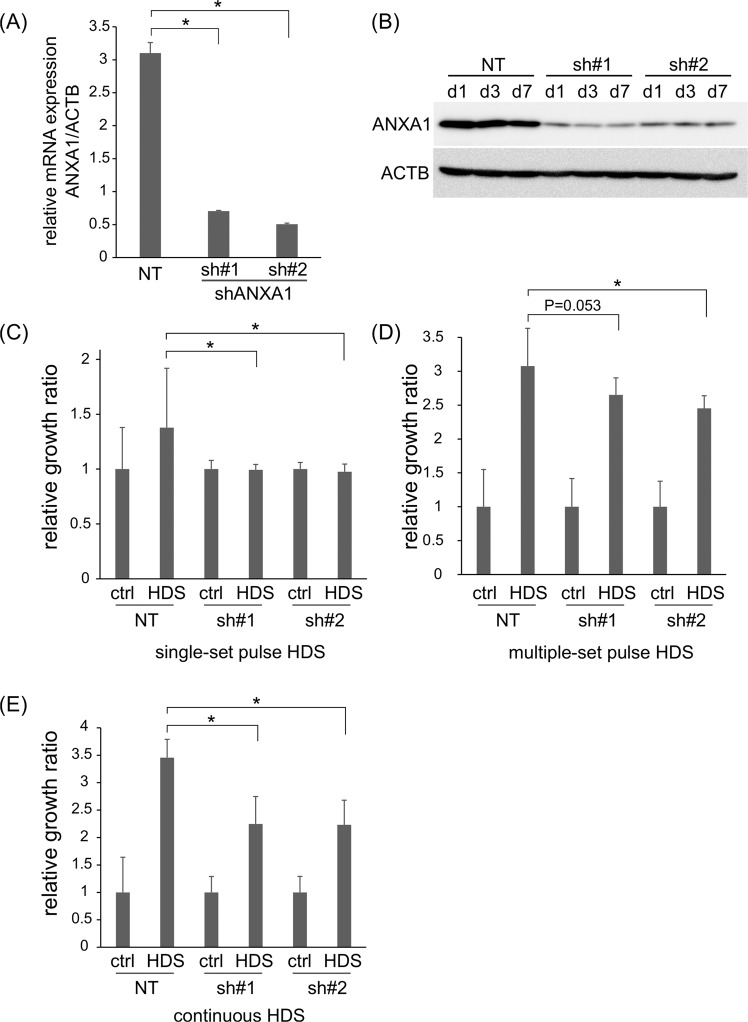
Knock down of *Annexin 1* (*ANXA1*) suppresses growth stimulated by hydrodynamic stress (HDS). **(A**) Semi-quantitative PCR analysis confirms the knockdown of *ANXA1* gene expression. Relative *ANXA1* expression levels are shown in C45 CTOS that were either transduced with an ANXA1 shRNA construct (sh#1 or sh#2) or a non-targeting (NT) control construct. ACTB: β-actin, the internal control gene. Data are the average ± SD. *P < 0.05. **(B**) Immunoblots show proteins from C45 CTOS that were not transduced (NT) or transduced with 2 different ANXA1 shRNAs (sh#1 and sh#2). Proteins were extracted at 1, 3, and 7 days (d1, d3, and d7, respectively) after subculturing. (**C**) Relative growth (ATP assay) of C45 CTOS transduced with control (NT) or ANXA1 shRNAs (sh#1 and sh#2) and cultured for 7 days after single-set pulse HDS. Data are the average ± SD, normalized to control (ctrl) values; N = 20 for each condition. *P < 0.05. **(D**,**E**) Relative growth (ATP assay) of C45 CTOS treated with control (NT) or ANXA1 shRNAs (sh#1 and sh#2), and cultured for 14 days with (**D**) multiple-set pulse HDS or with (**E**) continuous HDS. Data are the average ± SD, normalized to ctrl; N = 9 in (**D**) and N = 6 in (**E**) for each condition. *P < 0.05.

Next, we examined whether the upregulated ANXA1 expression induced by HDS was responsible for the growth stimulated by HDS. We constructed small-hairpin RNAs (shRNAs) that targeted ANXA1 (shANXA1s #1 and #2), and introduced them into the C45 CTOS line. Successful ANXA1 suppression was confirmed, both at the mRNA (Fig. 4A) and protein (Fig. 4B) levels. C45 CTOS growth stimulated by a single-set pulse HDS was abrogated by knocking down *ANXA1* expression (Fig. 4C). Similarly, the growth stimulatory effect of multiple-set pulse HDS was partially, but significantly suppressed by shANXA1 #2, while shANXA1 #1 showed a tendency to suppress the growth stimulation (Fig. 4D). Additionally, both shANXA1s #1 and #2 partially, but significantly suppressed the growth stimulatory effect of continuous HDS (Fig. 4E). Because ANXA1 functionally contributes to membrane repair, an ANXA1 knockdown might suppress the repair of membranes damaged by HDS exposure, and thus, affect the viability of cell clusters. However, we found that incorporation of FITC-dextran was not affected by the ANXA1 knockdown, suggesting that membrane damage level was not increased (Supplementary Fig. S9A). Also, cell viability at 1 day after HDS exposure was not altered by the ANXA1 knockdown (Supplementary Fig. S9B). Thus, the suppression of stimulated growth observed with the ANXA1 knockdown in C45 CTOS was not due to an increase in membrane damage or cell death.

**Figure 5:**
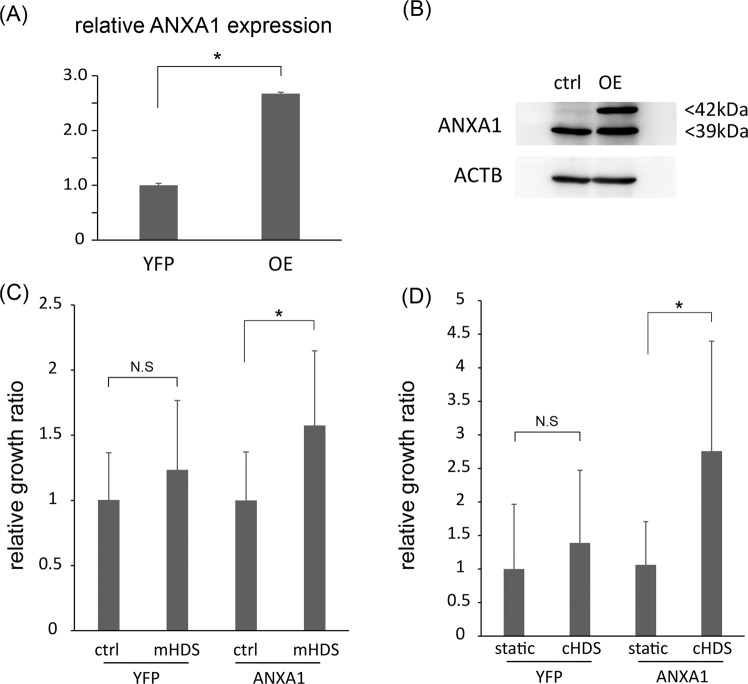
Forced overexpression of Annexin 1 (ANXA1) potentiates hydrodynamic stress (HDS)-induced growth stimulation in colorectal cancer cell clusters. **(A**) Semi-quantitative PCR analysis confirms *ANXA1* expression levels in C132 CTOS, transduced with either the ANXA1 construct (overexpression; OE) or the yellow fluorescent protein (YFP) control construct. Data are the average ± SD. *P < 0.05. (**B**) Immunoblots show ANXA1 expression in C132 CTOS transduced with ANXA1 (OE) or YFP (ctrl). The transduced ANXA1 had a higher molecular weight, because it was tagged with 3x FLAG. (**C**) Relative growth (ATP assay) of C132 CTOS with *ANXA1* OE and YFP as control. CTOSs were cultured for 14 days with multiple-set pulse HDS (mHDS) or without HDS (ctrl). Data are the average ± SD, normalized to YFP without HDS; N = 8 for each condition. *P < 0.05. (**D**) Relative growth (ATP assay) of C132 CTOS with *ANXA1* OE and YFP as control. CTOSs were cultured for 7 days with continuous HDS (cHDS) or without HDS (ctrl). Data are the average ± SD, normalized to control; N = 6 for each condition *P < 0.05.

Next, we focused on the C132 CTOS line. In this line, HDS did not induce significant growth stimulation, but it did induce ANXA1 expression (Figs. 3C and S8). We hypothesized that there might be an ANXA1 threshold level, which must be crossed to observe HDS stimulated growth, and that this particular CTOS line had ANXA1 levels that did not exceed the threshold. Therefore, we forced ANXA1 overexpression by transducing an exogenous ANXA1 expression vector into C132 CTOS cells (Fig. 5A,B), and we evaluated the growth stimulatory effect of HDS. The results showed that C132 growth was stimulated by multiple-set pulse HDS and continuous HDS, but only in C132 with overexpressed ANXA1, not in controls transduced with yellow fluorescent protein (YFP; Fig. 5C,D). These results supported the notion that ANXA1 contributed to HDS-induced growth stimulation.

### ANXA1 regulates the growth stimulatory effect by activating the PI3K/AKT pathway

**Figure 6:**
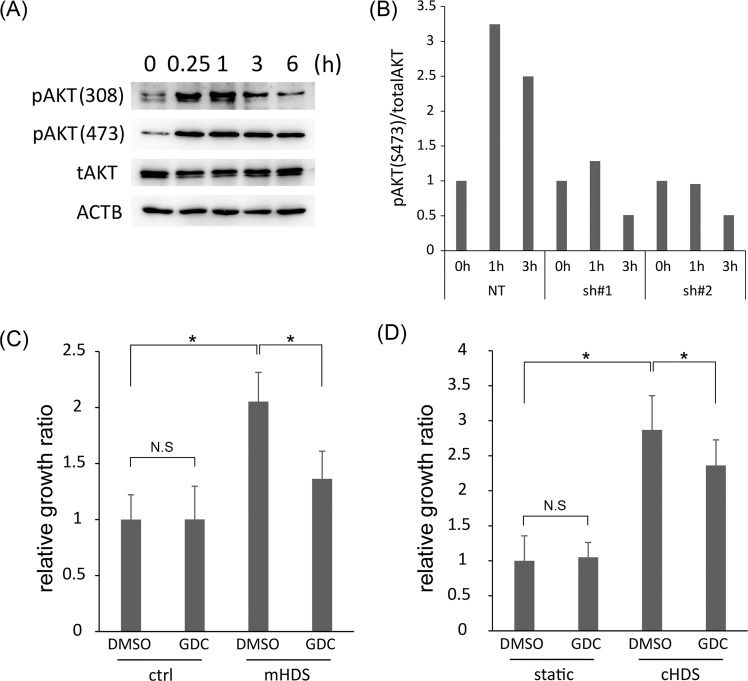
Hydrodynamic stress (HDS) stimulates growth by activating the PI3K/AKT pathway downstream of Annexin 1 (ANXA1). **(A**) Immunoblot shows changes in the expression of proteins from the C45 line of cancer tissue-originated spheroids (CTOSs), treated with single-set pulse HDS. Proteins were identified by the indicated antibodies over the 6 h following HDS treatment. pAKT: AKT phosphorylated (at the indicated residue); tAKT: total AKT protein; ACTB: β-actin, the internal control gene. **(B**) Densitometry analysis of immunoblots shows the level of phosphorylated AKT (at S473), relative to the total AKT at each time point. The C45 CTOS line was treated with control (NT) or ANXA1 shRNAs (sh#1 or sh#2), then single-set pulse HDS were applied. Protein samples were extracted at the indicated time points following HDS treatment. Data show the quantification of a single representative result, selected from 3 independent experiments (see Supplementary Fig. S10 for raw immunoblots). **(C**) Relative growth (ATP assay) of C45 CTOS, treated with 2.5 μM GDC-0941 (GDC), and cultured for 14 days with multiple-set pulse HDS (mHDS). Data are the average ± SD, normalized to the DMSO control (crtl); N = 8 for each condition. *P < 0.05. **(D**) Relative growth (ATP assay) of C45 CTOS, treated with 1 μM GDC-0941 (GDC), and cultured for 14 days with continuous HDS (cHDS). Data are the average ± SD, normalized to the DMSO control; N = 6 for each condition. *P < 0.05.

Finally, we evaluated the signaling pathway downstream of ANXA1. We previously reported that mechanical disruption of the CRC CTOS architecture by strong HDS could induce AKT phosphorylation through NRG/ERBB3^13^. In addition, ANXA1 could induce AKT phosphorylation by via activating PI3K in many types of cells^22^. In the C45 CTOS line, a single-set pulse HDS stimulated AKT phosphorylation, at both T308 and S473 (Fig. 6A). In C45 CTOSs with the ANXA1 knockdown, AKT phosphorylation was suppressed, particularly at S473 (Figs. 6B and S10). Next, to confirm the contribution of PI3K/AKT signaling to HDS-induced growth stimulation, we applied an FDA-approved, small compound PI3K inhibitor, GDC-0941, to the C45 CTOS culture with HDS. Although 1 μM GDC-0941 had no inhibitory effect on CTOS growth without HDS, GDC-0941 significantly suppressed the growth stimulated by multiple-set pulse HDS (Fig. 6C) and continuous HDS (Fig. 6D). This suppression was similar to the level of suppression observed with ANXA1 knockdowns (Fig. 4D,E). Taken together, these results indicated that ANXA1 contributed to HDS-induced growth stimulation in cancer cell clusters by activating the PI3K/AKT pathway.

## Discussion

In this study, we showed that cell clusters exhibited growth stimulation in response to HDS in a portion of CTOS derived from colorectal cancer. In addition, we demonstrated that ANXA1 expression and subsequent PI3K/AKT pathway activation comprised one of the underlying mechanisms.

Cancer cells are exposed to various HDS levels, depending on local conditions. Fluid shear stress is the most robustly studied type of HDS in cellular biology; the degree of fluid shear has been calculated and simulated in a physiological context. Interstitial fluid shear forces in the tumor at primary or metastatic sites range from 0 to 0.1 dyne/cm^2^ ^23^. On the other hand, fluid shear forces inside blood and lymphatic vessels are higher; the ranges are 4~30 dyne/cm^2^ in the artery, 1~4 dyne/cm^2^ in the vein, and 0~0.64 dyne/cm^2^ in the lymphatic vessels^23,24^. Maximum fluid shear forces can reach 3000 dyne/cm^2^, in some situations, such as at the bifurcation of a large artery^23,25^. In this study, cell clusters in medium were passed through needles (27 G, inner diameter: 0.22 mm) at a maximum rate of 15 ml/min. The estimated viscosity of the suspensions was 0.0078 Poise^26^. The maximum shear stress under these conditions, calculated with Poiseuille’s equation^27^, was 1866 dyne/cm^2^, near the wall; less shear stress was imposed inside the needle, which indicated that the present study was performed at stress levels that were physiologically plausible.

Although *in vitro* investigation of HDS in floating cancer cells, but not in adherent cells, is a relatively new area, researchers have developed HDS methods appropriate for each model. For a transient HDS exposure, cells were passed through a syringe and needle^27,28^ as was employed for our single-set and multiple-set pulse HDS. This simple method is useful for high-level transient HDS, but not suitable for continuous exposure of low-level HDS. To accurately simulate continuous low-level HDS, a peristaltic pump in a circulatory system was used^2,4,29^. Some researchers combined it with microfluidic devices. Alternative method for continuous low-level HDS is to generate laminar flow by rotating parallel-plate or cone-and-plate viscometer in a culture dish^30^. On the other hand, the requirement of these specialized complex devices makes it difficult to compare multiple conditions at the same time. As a more convenient method for comparing multiple conditions, culture vessels were simply swirled or rotated in an incubator^5,7,30^. We applied the rotation method for “continuous HDS” in the present work. The appropriate method should be selected considering the advantages of each method.

CTCs, defined as cancer cells detected in the blood system, acquire various survival advantages, and thus, they are suitable for metastasis^31^. Recent studies have suggested that CTCs in cell clusters (CTC-clusters) are more important than single CTCs, because CTC-clusters are much more viable, thus, they are responsible for metastasis^15^. Indeed, cancer cells derived from patients are more prone to anoikis when they are dispersed into single cells^12^ compared to those in established cell lines. Moreover, in a study that used established cancer cell lines, HDS induced death in single cells, even at very low shear stress levels (1–2 dyne/cm^2^)^32^. Despite this evidence that supports the significance of CTC-clusters, no report has described the effect of HDS on CTC-clusters. We previously reported that cancer cells can exist as polarlized cell clusters in blood and lymphatic vessels^33^. In the present study, we used CTOSs as a model of cancer cell clusters, and we showed that HDS promoted the growth of cell clusters. This finding might provide an additional explanation for findings in previous studies, where CTC clusters were shown to be more responsible for metastasis than single CTCs^15^. Further studies are expected to elucidate the contribution of HDS to the metastatic abilities of CTC-clusters.

Annexins are Ca^2+^ dependent, membrane-binding proteins that play important roles in the cell cycle, exocytosis, and apoptosis^20^. In addition, ANXA1 and ANXA2 and ANXA5 are involved in plasma membrane repair^20,21^. Injured membrane is repaired by forming patches via the fusion of intracellular compartments, and ANXA1 is recruited to those compartments^34^. ANXA1 also activates multiple signaling pathways, including the Src^35^, PI3K/AKT^36^, p38 MAPK^37^ and MEK/ERK^38^ pathways. Moreover, ANXA1 has multiple functional roles in promoting cancer phenotypes. In a pancreatic cancer cell line, ANXA1 played a role in preserving the malignant phenotype; it was involved in the potential for migration and invasion^39^. Additionally, ANXA1 was reported to be involved in resistance to 5-FU treatment, in CRC^40^, and to Trastuzumab treatment, in breast cancer^41^. Furthermore, investigations on ANXA1 expression in clinical cancer tissue specimens revealed a correlation between ANXA1 expression and poor prognoses in gastric^38^, bladder^42^, breast^22,41,43^, and renal^44^ cancers, which suggested that it played a role in regulating cancer progression. In the present study, we showed that the ANXA1/PI3K/AKT axis played a role in regulating the promotion of CRC CTOS growth in response to HDS. This was a novel role for the ANXA1/PI3K/AKT pathway in cancer biology.

The results presented in this study clearly indicated that HDS stimulated the growth of a certain population of CRCs. However, HDS-induced growth was not evident in all the CTOS lines examined. One potential explanation is that KRAS mutations might contribute to the response to HDS. In KRAS mutant CTOS lines, CB3, C45, and C75, both multiple-set pulse HDS and continuous HDS stimulated their growth. On the other hand, in KRAS wild-type CTOS lines, C111 and C132, neither multiple-set pulse HDS nor continuous HDS stimulated growth. C166, another KRAS wild-type CTOS line, acquired growth stimulation only with multiple-set pulse HDS, but not with continuous HDS. In addition, previous studies have shown a correlation between KRAS mutations and ANXA1 overexpression^45^. Conversely, ANXA1 acts directly on the EGFR to regulate the EGFR/Ras pathway. ANXA1 binds to the EGFR adaptor protein, Grb2, which increases Ras activity^46^. Those findings supported the hypothesis that KRAS contributed to the ANXA1-mediated growth stimulation induced by HDS. Future testing on more CTOS lines could clarify this potential correlation between mutant KRAS and HDS-induced growth.

The HDS-induced growth stimulation was not completely suppressed by knocking down ANXA1 or by inhibiting PI3K; moreover, it was independent of ANXA1 in the CB3 CTOS line. These results indicated that factors other than ANXA1 must be involved in the promotion of growth by HDS. Future studies on the contribution of HDS to pro-metastatic features of cancer cell clusters, e.g. stemness and invasiveness both *in vitro* and *in vivo*, could increase our understanding on the mechanisms of metastasis mediated by circulating cancer cell clusters.

## Materials and Methods

### CTOS preparation and maintenance

The study was approved by the Institutional Ethics Committees at Osaka International Cancer Institute, formerly known as Osaka Medical Center for Cancer and Cardiovascular Diseases, and Kyoto University. CTOSs were prepared from mouse xenografts, as described previously^12^. CTOSs were cultured in StemPro hESC medium (Invitrogen, Carlsbad, CA). For *in vitro* maintenance and expansion, CTOSs were subcultured once a week by syringe disruption method; CTOSs were passed through a 1-ml syringe with a 27 G needle at high flow rate (~30 ml/min), which disrupted them into multiple smaller fragments. CTOSs re-formed from these fragments were transferred to new dishes with fresh medium to start new culture^13^. For all assays, we used CTOSs at 3 days or more after subculture to avoid the influence of the subculturing procedure. Animal studies were approved by the Institutional Animal Care and Use Committee of Osaka International Cancer Institute and performed in compliance with institutional guidelines. To expand CTOSs after gene manipulation, CTOSs in Matrigel (BD Biosciences, Bedford, MA) were injected subcutaneously into NOD/SCID mice (CLEA Japan, Shizuoka, Japan) to generate xenograft tumors.

### Introduction of hydrodynamic stress to CTOSs

For single-set pulse HDS (Supplementary Fig. S1A), CTOS suspensions were passed through a syringe and needle with a syringe pump (YSP-301, YMC, Japan), as described elsewhere^27^, with a slight modification. Briefly, CTOSs were suspended in StemPro medium and loaded into a syringe with a 1-in 18 G needle manually at ~6 ml/min to minimize HDS. Then, the CTOS suspension was passed through a 3/4-in 27 G needle with a syringe pump, at the flow speed indicated in figure legends. This procedure of cycling, i.e., loading the CTOS suspension and passing it through a syringe, was repeated for the indicated times in figure legends; each cycle is referred to as a ‘syringe-passing’. In control conditions for single-set pulse HDS, CTOS suspension was passed through a 1-in 18 G needle, manually, at ~6 ml/min.

For multiple-set pulse HDS (Supplementary Fig. S1B), equal volumes of a CTOS suspension were dispensed into 24-well, non-treated plates (IWAKI, Japan). The pulse HDS were introduced by manually passing CTOS suspension through a 1/2-in 27 G needle attached to 1-ml syringe, at ~15 ml/min, and repeated 4 times. Seven sets of HDS pulses were given to cells during 2 weeks of culture (Supplementary Fig. S1B). In control conditions for multiple-set pulse HDS, CTOSs were cultured in 24-well plates without HDS exposure.

For continuous HDS (Supplementary Fig. S1C), equal volumes of the CTOS suspension were dispensed into 15-ml Bio-reaction tubes (CELLTREAT, Pepperell, MA), which have a vent-cap with a hydrophobic membrane that allows gas exchange. The cell culture medium was added up to 5 ml, and tubes were placed on a tube roller (MX-T6-S, DLAB Scientific, China) at ~15 rpm for 7 days, in a CO_2_ incubator (Supplementary Fig. S1C). For the continuous HDS control assay, CTOSs were dispensed into Bio-reaction tubes and remained static in the same incubator for 7 days.

The PI3K inhibitor, GDC-0941 (S1065, Selleck, Houston, TX), was dissolved in DMSO and used as indicated.

### Membrane damage assay

To chemically induce membrane damage, CTOSs were treated with SLO (01-531, Bio Academia, Japan), at the indicated concentrations, in StemPro medium for 10 min. Then, cells were washed with PBS, and suspended in fresh StemPro medium. Next, CTOSs, in StemPro medium, were dispensed into 24-well or 96-well plates for subsequent assays. Membrane damage was visualized by adding 62.5 μM FITC-dextran (4000 Da, 46944, Sigma, St. Louis, MO), which was introduced to CTOSs, concomitant with either the HDS or the SLO.

### CTOS growth assay

One day after starting the CTOS cultures for each assay, viable cells were quantified with the Realtime Glo assay (G9711, Promega, Madison, WI). Then, after the indicated time periods, we evaluated CTOS viability with CellTiter Glo (G7570, Promega, Madison, WI). Finally, we normalized these values to the luminescence intensity values measured with the RealTime Glo assay on day 1. Additionally, CTOS growth was evaluated by the size increase using photo imaging^47^, which is shown as “area growth” in Supplementary Fig. S3. CTOSs were prepared and cultured at ~10 CTOSs per well in a 96-well plate, and pictures of entire well were captured at day 0 and 7. CTOS area was measured and average calculated for each well, and the growth was evaluated as relative increase of the area from day 0 to 7. Cell3iMager duos (SCREEN, Japan) was used for the imaging and the measurement of the CTOS area.

### Semi-quantitative real-time PCR

RNA was extracted with the RNAeasy mini kit (Qiagen, Hilden, Germany). Reverse transcription was performed with Super Script III reverse transcriptase (Thermo Fisher Scientific, Waltham, MA), according to manufacturer instructions. Semi-quantitative real-time PCR was carried out with the StepOneReal-Time PCR System (Applied Biosystems, Foster City, CA), as described previously^48^. The relative expression of ANXA 1 is calculated by the 2(−delta delta Ct) method using ACTB for normalization. Data are presented as the mean ± SD of three replicates. We used the following primers: human β-actin (ACTB) F: cctggcacccagcacaat; human ACTB R: gccgatccacacggagtact; human ANXA1 F: gcaggcctggtttattgaaa; human ANXA1 R: gctgtgcattgtttcgctta.

### Immunoblotting

Western blotting was carried out as described previously^48^. The primary antibody against ANXA1 was obtained from Developmental Studies Hybridoma Bank (CPTC-ANXA1-1, Iowa, IA). The total AKT (#2920), pAKT-T308 (#9275), and pAKT-S473 (#4060) antibodies were obtained from Cell Signaling Technology (Danvers, MA). β-actin antibody was obtained from Sigma-Aldrich (St. Louis, MO). Densitometry was performed with ImageJ (NIH, Bethesda, ND).

### Microarray analysis

C45 CTOSs were collected, in duplicate, before and 6 h after exposure to single-set pulse HDS (3/4 in 27 G needle, 15 ml/min, 8 syringe-passings). RNA was extracted with an RNAeasy mini kit and DNAse I (Qiagen, Hilden, Germany) treatment. Microarray analysis was performed with the GeneChip Human Gene 2.0 ST Array. Signals were quantified and normalized with the RMA algorithm, as shown in Supplementary Table S1. At 6 h after HDS, 123 genes were upregulated by more than 1.5-fold. These genes were analyzed for GO enrichments with ToppFun (ToppGene Suite, https://toppgene.cchmc.org/)^49^. Microarray data from a previous study^13^ were also used to evaluate the overlap between HDS-induced genes and disruption-induced genes. At 6 h after disrupting C45 CTOS cells, 233 genes were upregulated by more than 5-fold. These data were analyzed to determine overlap with the 123 genes that were upregulated by HDS.

### Plasmid construction and gene transfer into CTOSs

Generation of constructs and electroporation was performed as previously described^50^. The pPiggyBac-Ubc.eGFP-Neo (pPB) and pCMV-hyPBase vectors were kind.pngts from Dr. Yusa (Wellcome Trust Sanger Institute, Cambridge, UK)^51^. For making short hairpin (sh) RNAs targeting ANXA1, we transferred the target sequences of the ANXA1 human shRNA constructs carried in pRS plasmids (TR314775, OriGene, Rockville, MD) to the pPB vector. The target sequences were as follows: shANXA1 #1: 5′-GCTATCCACAACTTCGCAGAGTGTTTCAG-3′ and shANXA1 #2: 5′-GGAACTCGCCATAAGGCATTGATCAGGAT-3′.

For ANXA1 overexpression assays, ANXA1 cDNA was cloned with the following primers: ANXA1 BamHI-F: cggatccATGgcaatggtatcagaattcc and ANXA1 stopdead MluI-R: cacgcgtgtttcctccacaaagagccac. The PCR product was cloned into the TOPO vector (Thermo Fischer Scientific), N-terminally 3x flag-tagged, and transferred into the pPB vector.

For electroporation, CTOSs were pretreated with 5 mM EDTA/PBS for 30 min at room temperature, followed by mixing with the pPB and pCMV-hyPBase vectors. Electroporation was performed in 2-mm gap cuvettes for 5 ms at 150 V, with a Type II NEPA21 electroporator (Nepa Gene, Chiba, Japan). After electroporation, CTOSs were selected for stable clones by neomycin (Roche Applied Science) and/or puromycin (Sigma-Aldrich).

### Statistical analysis

Significance was tested with the unpaired t-test, for single comparisons, and with the t-test and Bonferroni correction, for multiple comparisons. For the analyses of fluorescence intensity of FITC-dextran incorporated in CTOS, which did not show normal distribution, non-parametric tests were used: Wilcoxon rank sum test, for single comparisons, and Steel-Dwass test for multiple comparisons. P-values < 0.05 were considered significant.

## Supplementary information

Supplementary Information

Supplementary Information 2

SupplementaryInformation 3

SupplementaryInformation 4
