# Supplementary material for: Hydrodynamic stress stimulates growth of cell clusters via the ANXA1/PI3K/AKT axis in colorectal cancer
Source: Sci Rep. 2019 Dec 27;9:20027. doi: 10.1038/s41598-019-56739-7 (PMC6934682; doi:10.1038/s41598-019-56739-7)
Supplement: Supplementary file 1 — Supplementary Information [file 41598_2019_56739_MOESM1_ESM.pdf]

Supplementary Figures

Hydrodynamic stress stimulates growth of cell clusters via the ANXA1/PI3K/AKT axis in colorectal cancer

Takeshi Hagihara[1,2,3], *Jumpei Kondo[1,3], Hiroko Endo[3], Masayuki Ohue[3], Yoshiharu Sakai[2], Masahiro Inoue[1,3]

[1]Department of Clinical Bio-resource Research and Development, Graduate School of Medicine, Kyoto University, Yoshida-honmachi, Sakyo-ku, Kyoto 606-8501, Japan
[2]Division of Gastrointestinal Surgery, Department of Surgery, Graduate School of Medicine, Kyoto University, Yoshida-honmachi, Sakyo-ku, Kyoto 606-8501, Japan
[3]Department of Biochemistry, Osaka International Cancer Institute, 3-1-69, Otemae, Chuo-ku, Osaka 541-8567, Japan

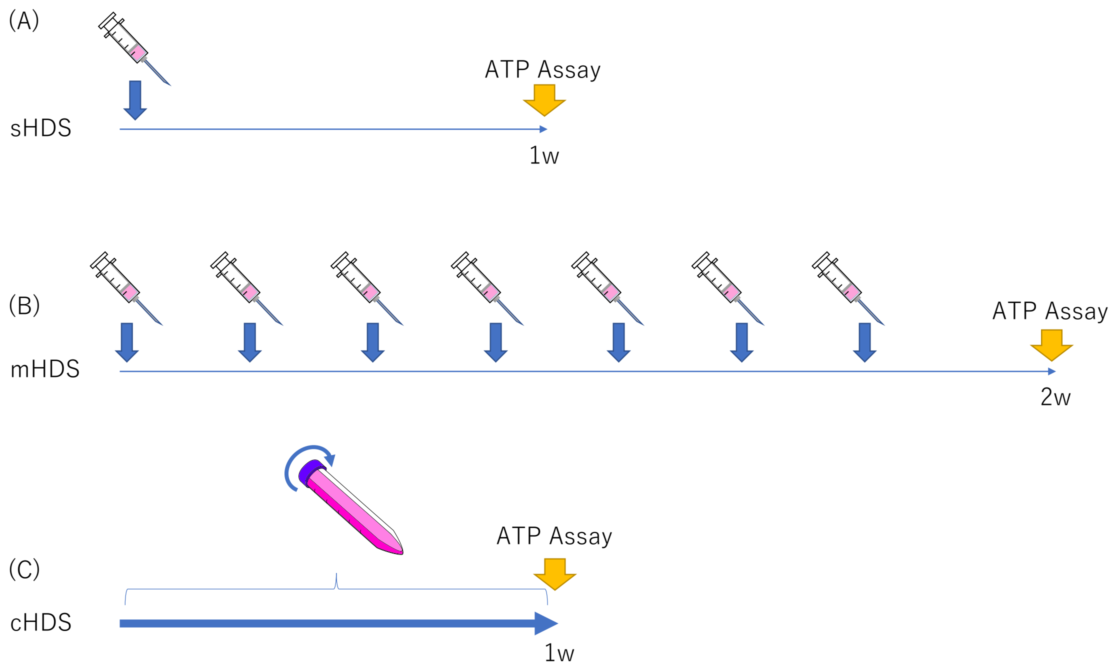

Figure S1

Schema of the experimental procedures for (A) single-set HDS pulses (sHDS), (B) multiple-set HDS pulses (mHDS) and (C) continuous HDS (cHDS). ATP assays were performed with Celltiter-Glo (Promega).

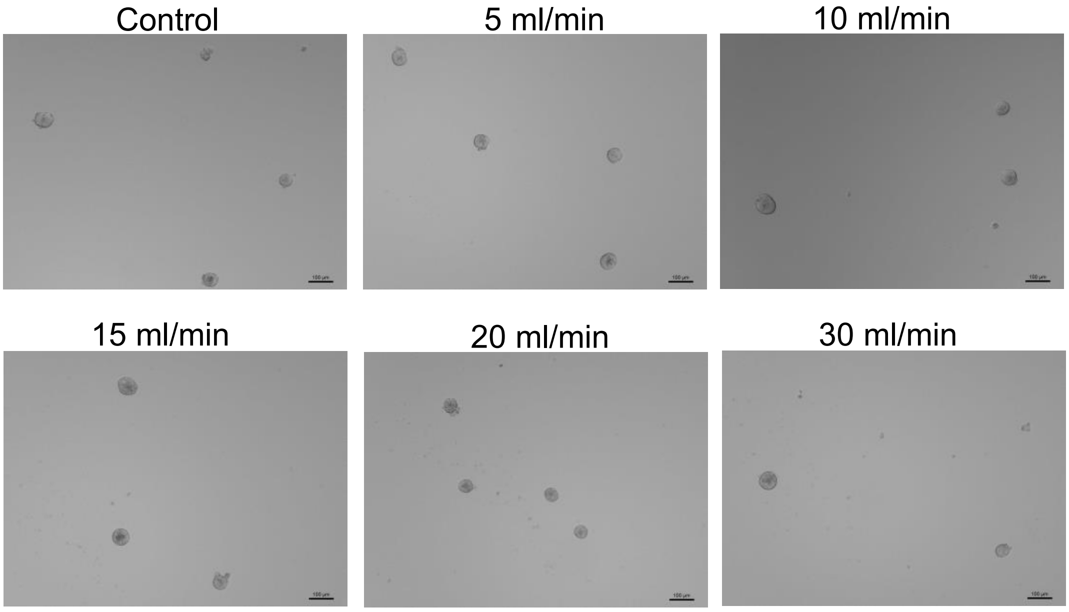

Figure S2

Bright-field images of C45 CTOSs, right after treatment with HDS, applied at the indicated flow rates. Scale bars: 100 μm.

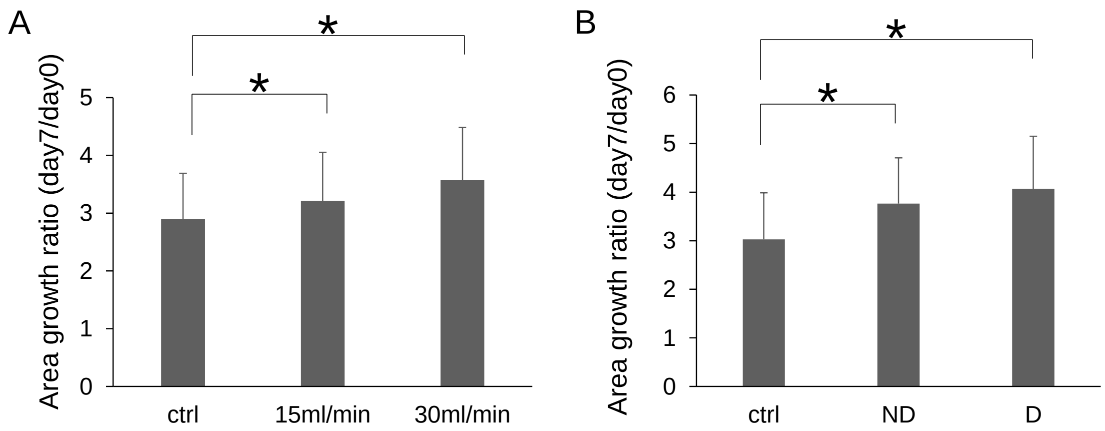

Figure S3

A. Area growth of C45 CTOS, 7 days after single-set pulse HDS (4 syringe-passings) delivered at different flow rates. The values are the average ± SD. N=20 for each condition; *P<0.05.

B. Area growth of C45 CTOS, with non-disrupted (ND) or disrupted (D) morphology, 7 days after a single-set pulse HDS. The values are the average ± SD. N=20 for each condition. *P<0.05.

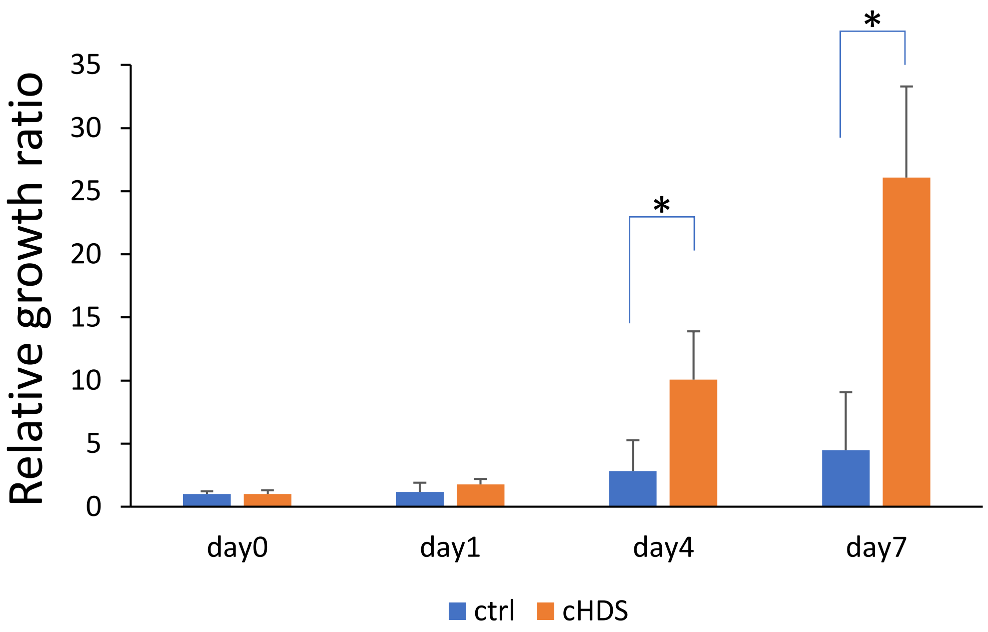

Figure S4

Relative growth (ATP assay) of C45 CTOS treated with continuous HDS (cHDS) over time. Data are the average ± SD, normalized to control (ctrl) at day 0; N=15 for each condition. *P<0.05

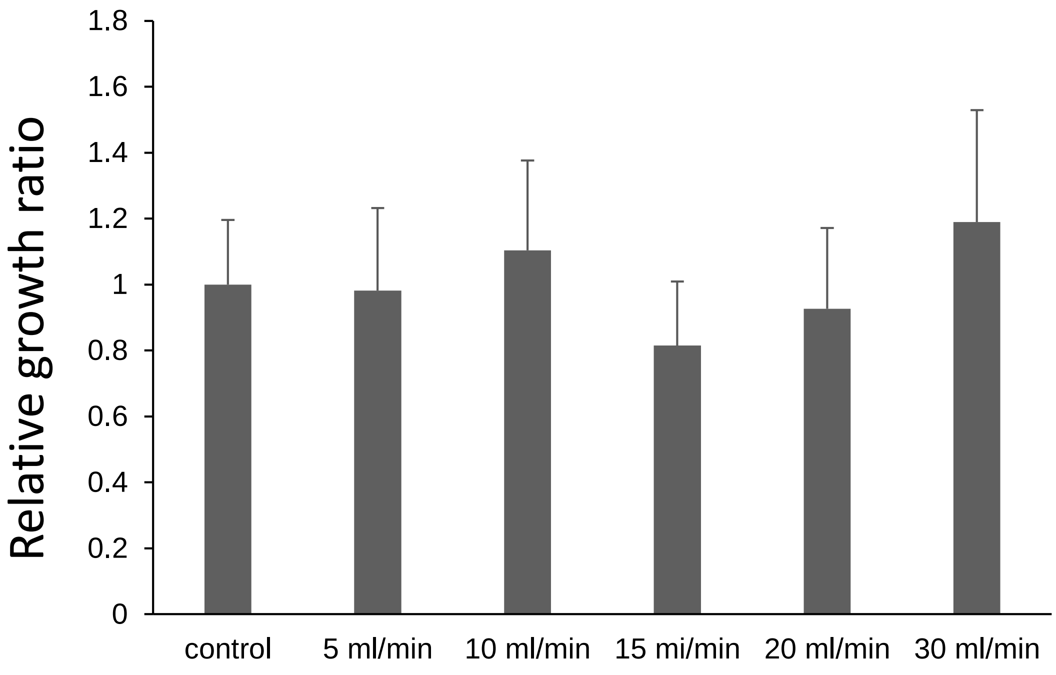

Figure S5

Relative growth (ATP assay) of single cells dispersed from C45 CTOSs, treated with single-set HDS pulses at the indicated flow rates. Data are the average ± SD, normalized to control; N=10 for each condition. No condition showed a significant change compared to control.

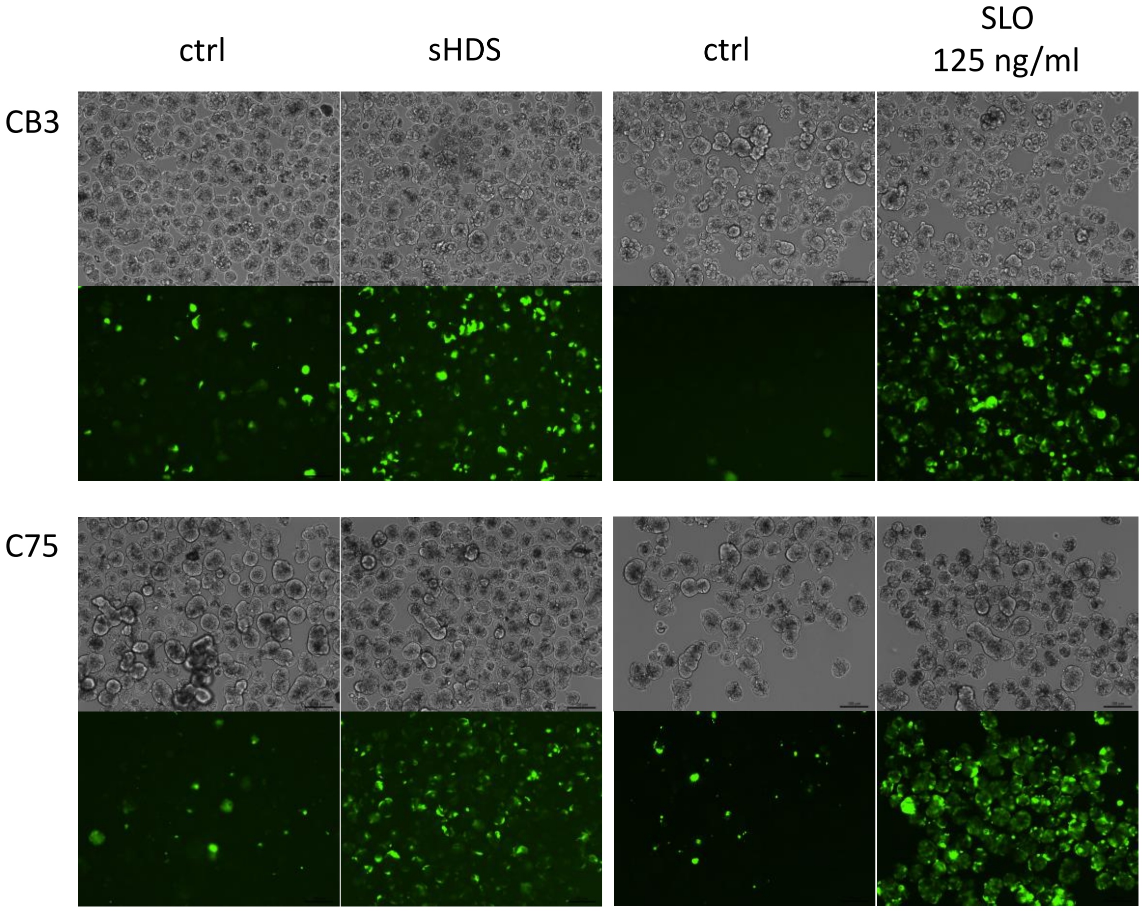

Figure S6

Fluorescence and bright-field images of CB3 and C75 CTOS lines, treated with either single-set HDS pulses (sHDS) or streptlysin-O (SLO), in the presence of FITC-conjugated dextran.

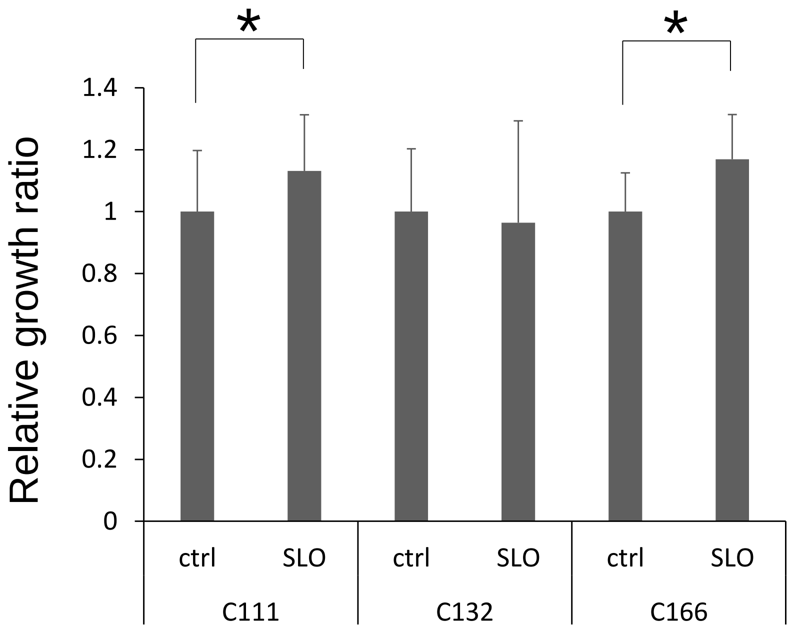

Figure S7

Relative growth (ATP assay) of C111, C132, and C166 CTOS lines, 7 days after treatment without (ctrl) or with 125 ng/ml streptlysin-O (SLO). Data are the average ± SD; N=20 for each condition. *P<0.05.

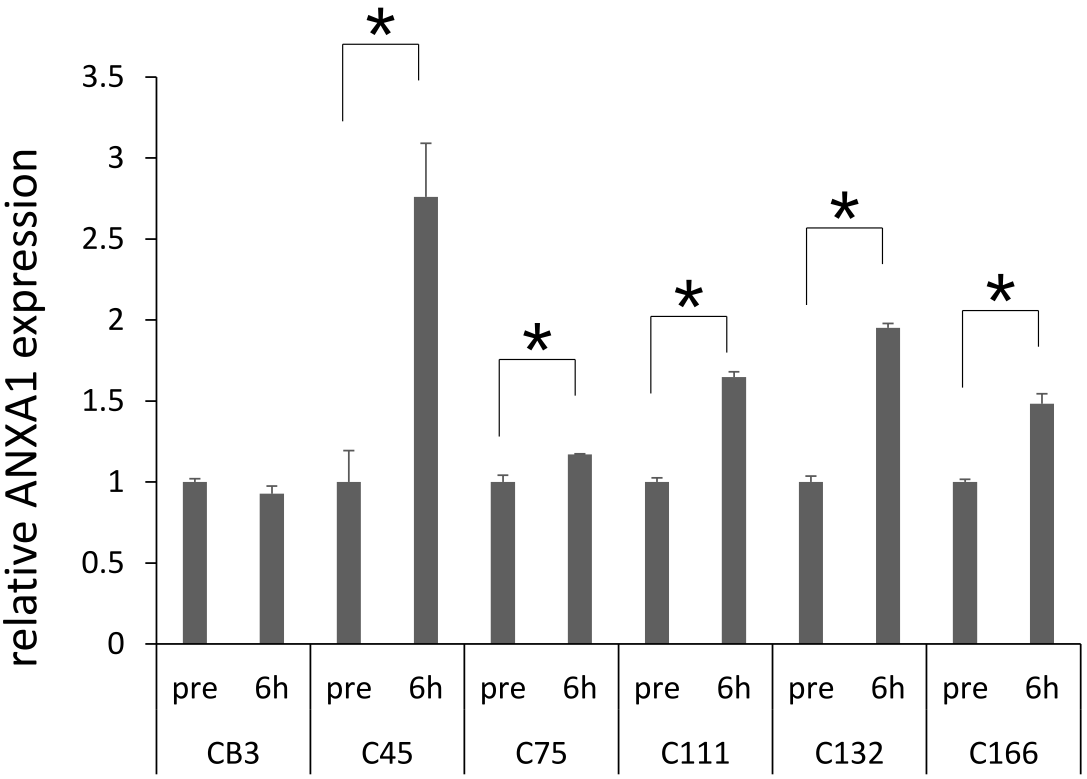

Figure S8

Relative expression of ANXA1 mRNA, measured before (pre) and 6 h after continuous HDS, in multiple CTOS lines. Data are the average ± SD; N=3 for each run. *P<0.05.

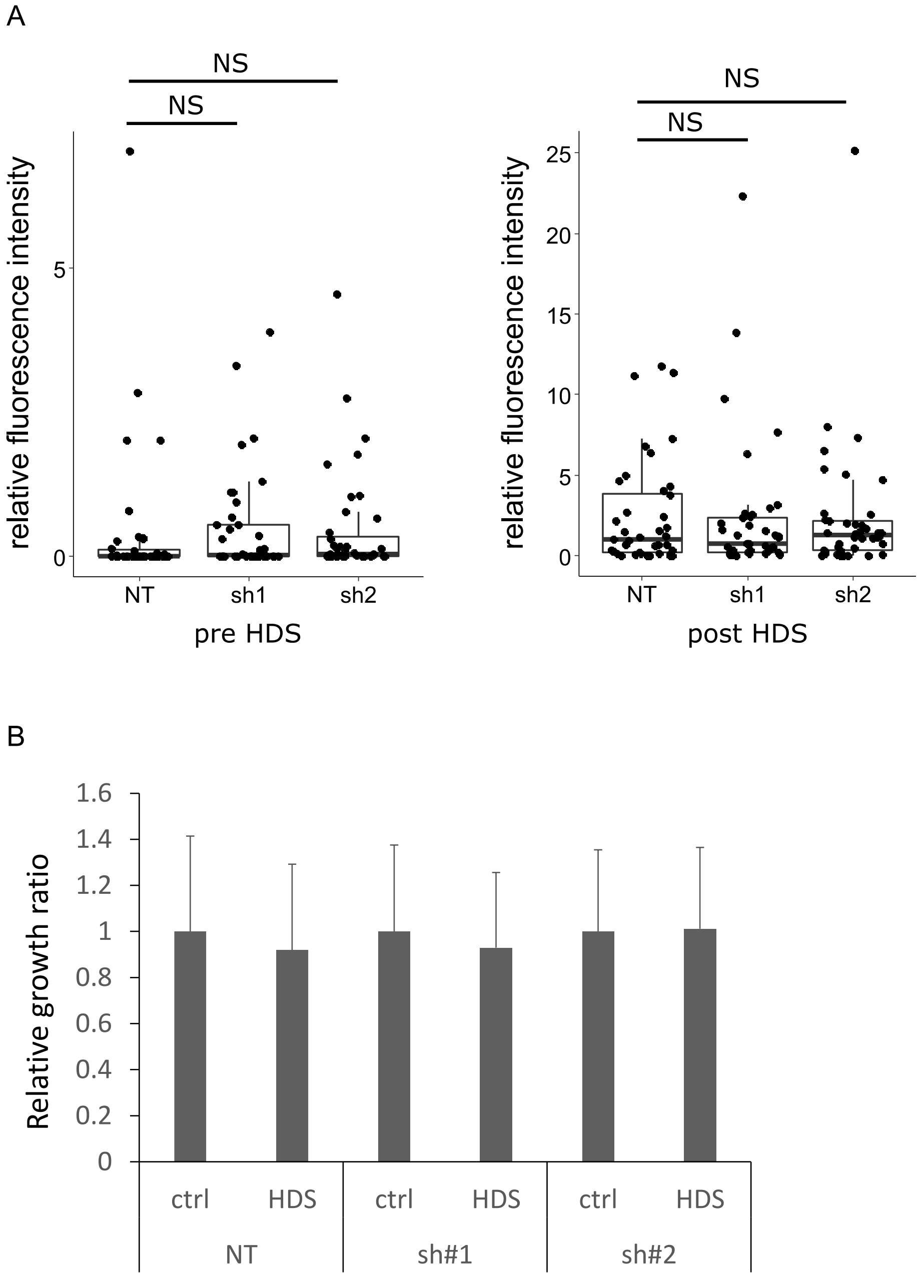

Figure S9

A. Quantified fluorescence intensity of CTOSs before (pre HDS) and after (post HDS) single-set pulse HDS. Data are the box-and-whisker dot plots; the boxes indicate inter-quartile range and the horizontal lines depict median, and the vertical lines show 1.5 times the inter-quartile range. Each dot is the intensity value of individual single CTOS, N=40 for each condition. Steel-Dwass test was performed for statistical analyses. NS: not significant.

B. Relative growth (ATP assay) of C45 CTOS, transduced with control (NT) or ANXA1 shRNAs (sh#1 or sh#2), measured at 1 day after single-set HDS pulses or no HDS (ctrl). Data are the average ± SD, normalized to each control (ctrl); N=30 for each condition. No condition showed a significant change compared to the corresponding control.

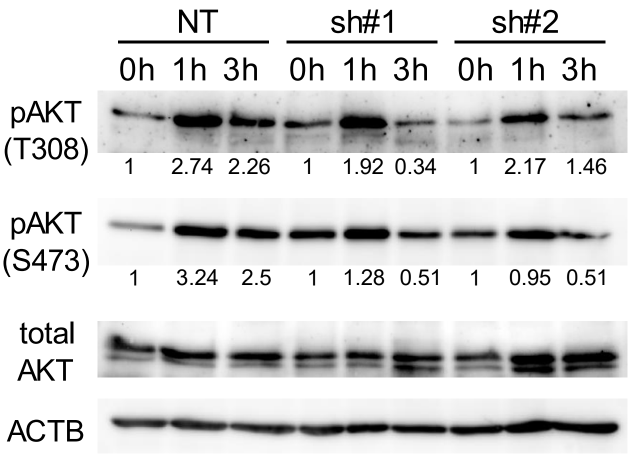

Figure S10

Immunoblots of proteins from C45 CTOS transduced with control (NT) or ANXA1 shRNAs (sh#1 or sh#2), then treated with single-set HDS pulses. Changes in phosphorylated AKT (pAKT) expression over time were measured with densitometry (values relative to total AKT are shown below each band). ACTB: β-actin, the internal control gene.

(Original blots for Fig. 4B)

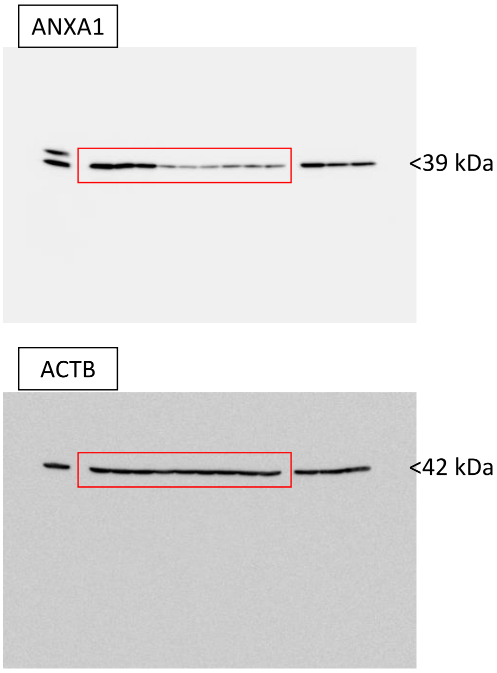

(Original blots for Fig. 5B)

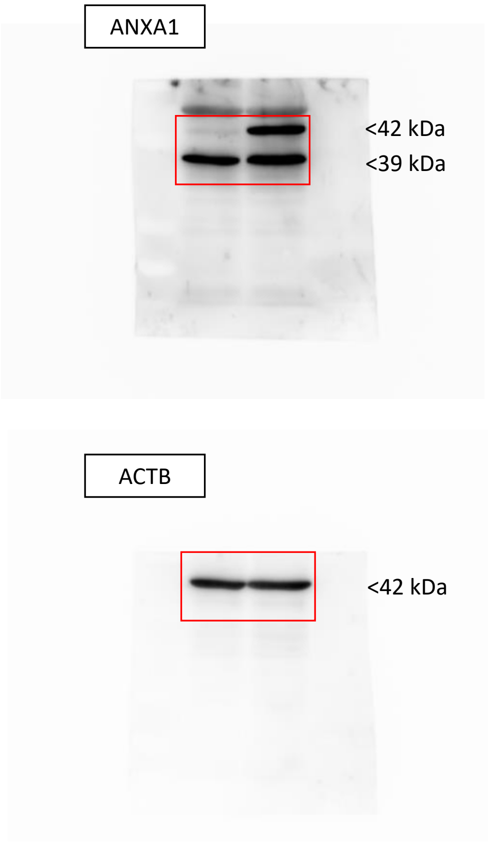

(Original blots for Fig. 6A)

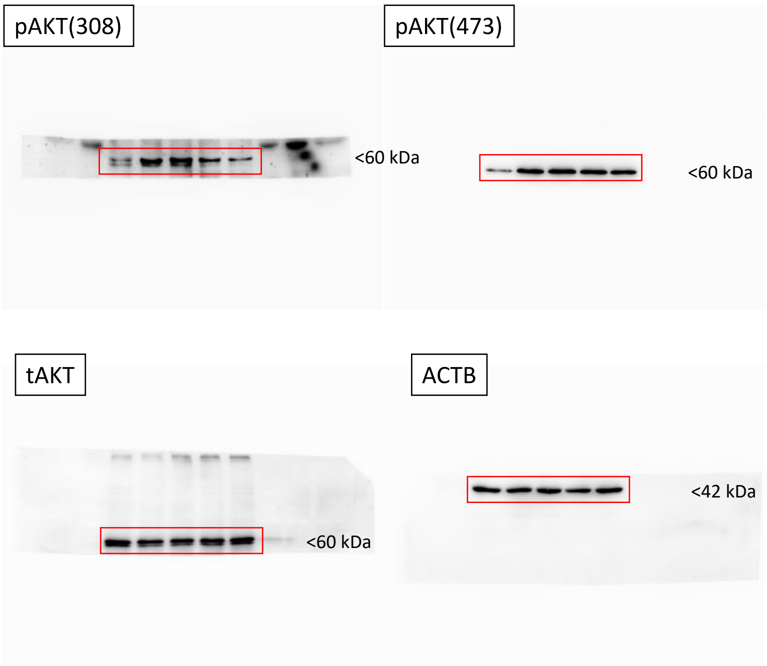

Figure S11

Original blots for immunoblot images presented in main figures. Areas enclosed with red boxes are cropped and used for main figures.
